# Corrigendum: Regulation of multiple carbon monoxide consumption pathways in anaerobic bacteria

**DOI:** 10.3389/fmicb.2018.01016

**Published:** 2018-07-05

**Authors:** Stephen M. Techtmann, Albert S. Colman, Michael B. Murphy, Wendy S. Schackwitz, Lynne A. Goodwin, Frank T. Robb

**Affiliations:** ^1^Institute of Marine and Environmental Technology, University of Maryland, Baltimore, MD, United States; ^2^Department of the Geophysical Sciences, University of Chicago, Chicago, IL, United States; ^3^GE Healthcare, Piscataway, NJ, United States; ^4^Department of Energy, Joint Genome Institute, Walnut Creek, CA, United States; ^5^Bioinformatics, Joint Genome Institute, Los Alamos National Laboratory, Los Alamos, NM, United States

**Keywords:** carbon monoxide, thermophiles, hydrogenogens, carboxydotrophs, *Carboxydothermus hydrogenoformans*, carbon monoxide dehydrogenase, CooA

In the original article, there was a mistake in Figure 2 as published. Specifically, panel 2D was included in error thus, the authors wish to remove it. Consequently, the caption for Figure 2 has been updated due to the removal of 2D, and the in-text citation has been removed from the following sentence: “*E. coli* overexpressing wild type CooA-2 acquired a red color similar to the color of CooA-1 expressing *E. coli* as described in Youn et al. (2004) due to the accumulation of the heme containing protein (Figure 2D)” in Results section, subsection “Expression and Characterization of CooA-2”. The corrected Figure 2 appears below.

**Figure 2:**
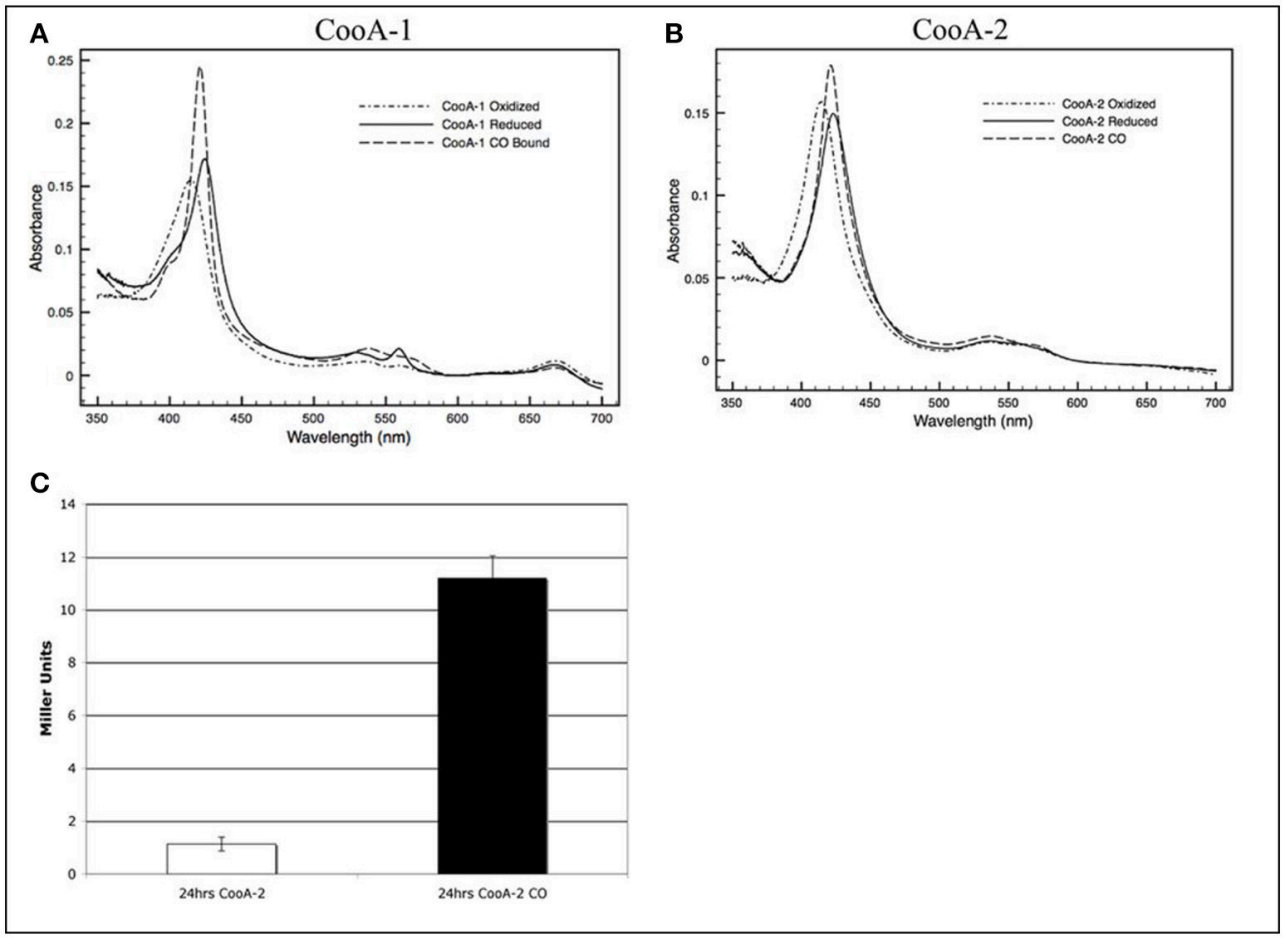
Visible spectra of a 2.2 μM **(A)** CooA-1 and **(B)** CooA-2. Dotted lines indicate the spectrum of oxidized CooA. Solid line indicates reduced CooA. Dashed line indicates reduced CooA under 1 atm of CO. **(C)** β-galactosidase activity of CooA-2 expressing *E. coli* DH5α with a *lacZ* under the control of the *R. rubrum cooF* promoter. Experiments with CO were grown with 2% CO in the headspace of the culture.

Additionally, there was a mistake in Figure 4. In the figure, the gel images had been edited. The no-protein control lane was moved to be adjacent to the relevant experimental lanes by removing empty lanes or ones with other controls. The authors neglected to mention this adjustment in the submission of the paper and wish to replace Figure 4 with an updated version where the spliced lanes have been clearly separated from the contiguous gel regions with black lines and white space to indicate where the control lanes are. The corrected Figure 4, as well as the original gel image used to prepare Figure 4, appear below.

**Figure 4:**
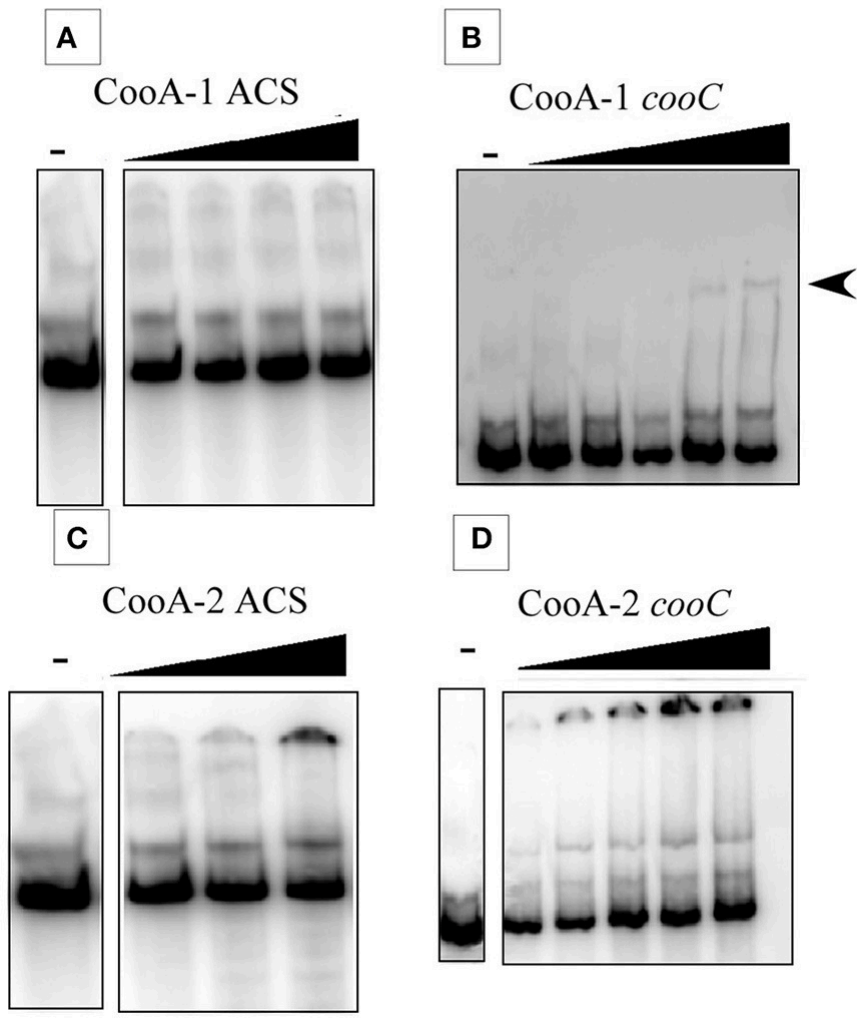
Electrophoretic mobility shift assays at 25°C. Gel shift results showing the binding of CooA-1 or CooA-2 to either p*acs* or the p*cooC*^*hyd*^ promters from *C. hydrogenoformans*. Arrows indicate formation of the CooA-promoter complex. **(A)** CooA-1 binding to p*acs* (Protein added: 3, 6, 12, 18 μg/ml). **(B)** CooA-1 binding to p*cooC*^*hyd*^ (Protein added: 3, 6, 9, 12, 15 μg/ml). **(C)** CooA-2 binding to p*acs* (Protein added: 3, 6, 9 μg/ml). **(D)** CooA-2 binding to p*cooC*^*hyd*^ (Protein added: 3, 6, 9, 12, 15 μg/ml).

**Figure:**
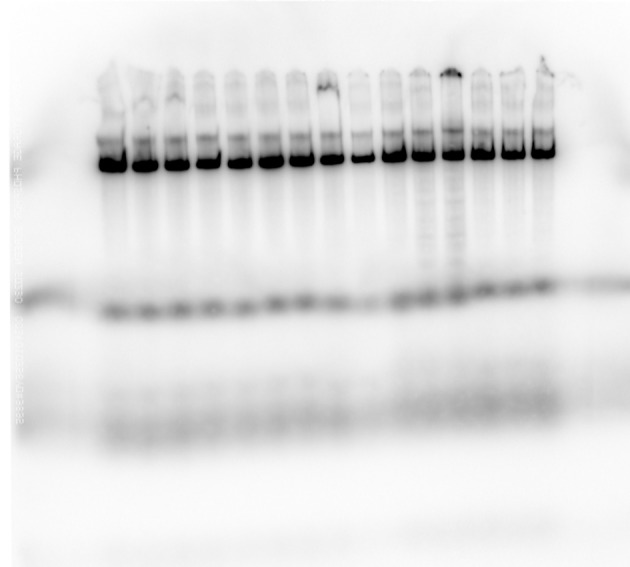
The original gel image used to prepare Figure 4.

The authors apologize for these oversights and state that these errors do not change the scientific conclusions of the article in any way.

The original article has been updated.

## Conflict of interest statement

The authors declare that the research was conducted in the absence of any commercial or financial relationships that could be construed as a potential conflict of interest.

